# Development of an Index of Engagement in HIV Care: An Adapted Internet-Based Delphi Process

**DOI:** 10.2196/resprot.8520

**Published:** 2017-12-05

**Authors:** Mallory O Johnson, Kimberly A Koester, Troy Wood, Torsten B Neilands, Jamie L Pomeranz, Katerina A Christopoulos

**Affiliations:** ^1^ University of California, San Francisco San Francisco, CA United States; ^2^ University of Florida Gainesville, FL United States

**Keywords:** HIV, AIDS, engagement in care, Delphi method, retention in care

## Abstract

**Background:**

Improving engagement in medical care among persons living with human immunodeficiency virus (HIV) is critical to optimizing clinical outcomes and reducing onward transmission of HIV. However, a clear conceptualization of what it means to be engaged in HIV care is lacking, and thus efforts to measure and enhance engagement in care are limited.

**Objective:**

This paper describes the use of a modified online Delphi process of consensus building to solicit input from a range of HIV and non-HIV researchers and providers, and to integrate that input with focus group data conducted with HIV-infected patients. The overarching goal was to generate items for a patient-centered measure of engagement in HIV care for use in future research and clinical practice.

**Methods:**

We recruited 66 expert panelists from around the United States. Starting with six open-ended questions, we used four rounds of online Delphi data collection in tandem with 12 in-person focus groups with patients and cognitive interviews with 25 patients.

**Results:**

We recruited 66 expert panelists from around the United States and 64 (97%) were retained for four rounds of data collection. Starting with six open-ended questions, we used four rounds of online Delphi data collection in tandem with 12 in-person focus groups with patients and cognitive interviews with 25 patients. The process resulted in an expansion to 120 topics that were subsequently reduced to 13 candidate items for the planned assessment measure.

**Conclusions:**

The process was an efficient method of soliciting input from geographically separated and busy experts across a range of disciplines and professional roles with the aim of arriving at a coherent definition of engagement in HIV care and a manageable set of survey items to assess it. Next steps are to validate the utility of the new measure in predicting retention in care, adherence to treatment, and clinical outcomes among patients living with HIV.

## Introduction

In 2011, researchers estimated that only 19% of individuals living with human immunodeficiency virus (HIV) in the United States achieve virologic suppression [1]. This finding led to renewed efforts to improve the steps leading up to this critical health outcome, including linkage to antiretroviral therapy initiation to ongoing retention in care [2-6]. Researchers and clinicians frequently use the term “engagement in care” to describe these steps; however, little clarity exists on what defines the engaged patient with respect to HIV care and treatment. For example, appointment attendance does not necessarily equal being invested in one’s care, although it is certainly a prerequisite. In addition, the benchmarks of the care cascade are not necessarily what is meaningful to patients as they move through the trajectory of care and treatment, and thus definitions of engagement must account for patient perspectives [7-9]. Finally, patients experience challenges in maintaining consistent involvement with HIV care and treatment over the life course, and patients may experience events that cause them to participate inconsistently or drop out altogether. A broader conceptualization of engagement in HIV care can guide the identification and measurement of relevant components of engagement in care. This, in turn, can permit the prediction of who is at risk for poor outcomes.

The literature describes a rich but dispersed set of factors likely related to engagement in care. For example, researchers have long established the importance of patient involvement in clinical decision making [10,11], with the conclusion that greater patient participation leads to greater satisfaction and adherence to care [10,12]. Studies have linked patient-provider relationship constructs to a wide range of proximal (eg, adherence to treatment and keeping appointments) [13] and distal outcomes (eg, virologic suppression and survival) in HIV care [12,14]. Moreover, patient reports of satisfaction, trust, perceived competence of providers, and the belief that a provider knows them as a person [15] associate with better HIV treatment adherence, retention in care, adaptive use of health care resources, and clinical outcomes over time [16-22]. Accordingly, such constructs may be critical components in the development of a patient-centered index of engagement in care. Acknowledging the value of patient, provider, and researcher perspectives on engagement in care, we designed a study to solicit and synthesize these points of view.

In this paper, we describe the use of an online Delphi method of consensus building [23-25], integrated with focus groups and cognitive interviews with patients, to identify content for a patient-centered measure of engagement in HIV care. The Delphi approach to consensus building has a long history in behavioral and health services research [26-30]. In this method, a group of experts complete a series of iterative questionnaires over multiple rounds, which begin with open-ended questions and conclude with more closed-ended items. Participants access the study materials for all rounds via a secure Web portal that maintains respondents’ confidentiality. Reponses from the first round of open-ended questions form the basis of the second round of questions, which are more specific and closed ended. This process is repeated, allowing respondents to review feedback on the collective responses of the group, until the group achieves consensus or agreement on the topic under investigation. Key elements of the process include protocols to maintain anonymity of the respondents’ identities and responses, multiple iterations of data collection, rapid analysis of responses, and feedback of the group’s collective responses at each successive round.

In this report, we describe the process used to identify key elements, components, and indicators of engagement in care to inform the development of a self-report measure of engagement in care to be administered to HIV-infected patients who are receiving HIV primary care. Specifically, we highlight the innovative use of Internet-based recruitment and data collection, coupled with in-person patient focus groups, to solicit input on a current challenge in HIV research. To our knowledge, this use of Internet-based Delphi methods, integrated with in-person focus groups with patients, is the first of its kind in the Delphi literature. A resulting definition of engagement in care along with specific items to assess it will offer a framework for greater understanding of engagement in care. This understanding can, in turn, provide guidance for interventions and policies to optimize engagement in HIV care with the added opportunity to apply the lessons learned and successful procedures learned during this investigation in future studies.

## Methods

### Design

**Figure 1:**
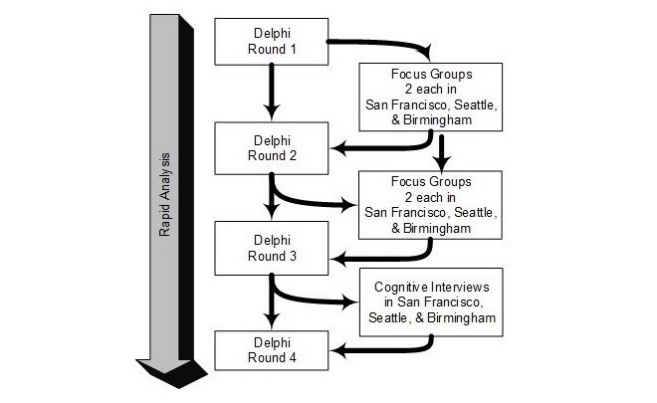
Interaction of Delphi, focus group, and cognitive interview methods.

We used four rounds of online Delphi data collection integrated with face-to-face patient focus groups that were conducted before Delphi rounds 2 and 3 in San Francisco, CA; Seattle, WA; and Birmingham, AL (see Figure 1 for the sequence of data collection procedures). Focus groups were conducted with patients having a range of retention in care profiles. Focus group participants were recruited through provider referral at a HIV specialty clinic in each of three cities: San Francisco, CA; Birmingham, AL; and Seattle, WA. Half of the groups in each city included only patients with optimal retention in the prior year (ie, no missed visits, no large gaps between visits) and the other half were less well-retained (ie, having any missed visits or greater than 6-month gaps between visits). Results from the focus group discussions were fed back into subsequent Delphi rounds. Specifically, patient perspectives that did not emerge from the Delphi process were integrated into subsequent Delphi rounds. The second round of focus groups also included an opportunity for patients to provide input on topics and candidate items generated up to that point. Between rounds 3 and 4, we conducted cognitive interviews with patients of the candidate survey items at the three primary research sites. Specific findings from the focus groups are forthcoming; this paper focuses on the Delphi process. All Delphi procedures, patient focus groups, and cognitive interview procedures were approved by the Committee for Human Research at the University of California, San Francisco. In addition, focus group and cognitive interview procedures were approved by our partnering institutions’ institutional review boards at the University of Washington in Seattle, WA, and the University of Alabama at Birmingham in Birmingham, AL.

### Identification, Recruitment, and Enrollment of Delphi Panel Members

We identified three subgroups of experts to include in the Delphi panel (20 each for a target total of 60). We identified group 1 (HIV researchers) by using NIH Reporter, PubMed, and Patient-Centered Outcomes Research Institute (PCORI) grantee lists to identify investigators who had published on HIV care and treatment engagement in the past 5 years or were currently funded to work in the area. Using a range of keywords (eg, retention in care, engagement in care), we identified a diverse group of investigators with regard to geography and training background (eg, clinical, behavioral, and epidemiological scientists). We recruited group 2 (HIV providers with at least half-time clinical practice) through email invitations from our partners in the Centers for AIDS Research Network of Integrated Clinical Systems (CNICS), a cohort of more than 30,000 patients at eight HIV care sites in the United States. For this group, we sought a range of providers, including physicians, nurse practitioners, physician assistants, nurses, and social workers. For group 3 (non-HIV providers and researchers), we sought to broaden input beyond HIV by enrolling panelists who were working outside of HIV, both in research in the area of engagement in care and clinical care delivery in high-volume primary care. We located these respondents through a similar approach as group 1: searching for publications on the topic of engagement in care in other chronic conditions and through local contacts. We used these resources and referrals from those recruited to groups 1 and 2 to identify clinicians (with at least half-time clinical practice) working outside of HIV with high volumes of patients.

While maintaining approximately equal proportions across these three groups, potential panelists were approached via an email from the principal investigators of the study (cosigned by the NIH Program Officer), which briefly explained the purpose of the study, the expected time commitment, and the compensation for participation. We directed those who agreed to a secure website, where the instructions guided them through the consent process and a brief survey to collect information to characterize the participants (eg, age, race, ethnicity, gender, academic discipline, clinical practice characteristics, years since completing degree/training). Once we achieved the targeted sample size, we initiated the first round of data collection. To incentivize enrollment and improve retention among the panelists, we offered a US $50 per round electronic gift card payment with a bonus of an additional US $50 to panelists who completed all four rounds.

### Delphi Survey Procedures

We used Qualtrics (Provo, UT, USA), a software program that allows the building, distribution, and analysis of online surveys for administration of the online Delphi surveys. Participants were sent a unique URL directing them to each round of the survey, and reminder emails were sent as the deadlines approached. Each round was open for 3 weeks with some extensions (up to a week) provided when requested. We chose four rounds on Delphi data collection because this was in line with the most common number of rounds identified in the Delphi literature [23-25].

### Content of the Delphi Surveys

The objectives of the online Delphi surveys were to solicit patient-centered constructs that are relevant to engagement in care and to review and comment on patients’ perspectives on engagement in care. The survey began with demographics and six open-ended items to encourage a wide range of responses (round 1). These included:

1. How would you describe patients who are well engaged in health care?
2. How would you describe patients who are not well engaged in health care? Please elaborate as much as possible and feel free to use examples.
3. What clinic, provider, and other factors influence patient engagement in health care?
4. Overall, what factors do you think are not emphasized enough with regard to patient engagement in health care?
5. The HIV research and treatment field generally defines the well-engaged patient as a someone who comes to all medical appointments and takes HIV medications on a consistent basis, meaning not missing doses. Please discuss your thoughts about this definition.
6. What else should we be considering with regard to patient engagement in health care (that we haven’t already covered in the preceding questions)? Why?

Each subsequent round of Delphi included increasingly specific content, such that round 2 had all items that were identified as potentially relevant to engagement in HIV care. We then asked Delphi panelists to rate each of them along a scale created for this study with anchor points from 0 (not at all important to engagement in care) to 100 (extremely important to engagement in care). Round 2 also allowed panelists the opportunity to offer comments or wording recommendations for the items.

Prior to round 3, analysis focused on two general goals: (1) to develop a working definition of the concept of engagement in care, and (2) to reduce the number of items for the planned new self-report measure (see subsequent Delphi Analysis section). In round 3, we asked experts for feedback on the definition of engagement in care and whether we should include each item in the planned self-report survey instrument to be administered to patients. Panelists rated the items along the following scale from 0 to 100: 0 (absolutely do not include), 50 (maybe include if you have space), 100 (absolutely include). We then again solicited suggestions for wording revisions.

Prior to round 4, we took the most important topics from round 3 by creating items through intensive team discussion, identifying response choices, and conducting individual cognitive interviews with patients at each of the three sites (25 patients total). A resulting small number of items were retained and shared with the panelists in round 4. In that final round, panelists were asked how much they thought each item might predict three key outcomes (adherence to medications, retention in care, virologic suppression) using a five-point Likert scale (not at all, a little, a moderate amount, a lot, and a great deal). Round 4 also included an opportunity to provide feedback on the Delphi study methodology from the panelists.

### Delphi Analysis

The investigative team conducted qualitative data analysis of the round 1 Delphi results and the focus groups under the direction of an experienced qualitative researcher. We analyzed responses to the Delphi rounds using content analysis, a standard qualitative technique for cataloging open-ended survey questions [31,32]. This technique is useful for analysis of qualitative data when some a priori domains are defined based on the research questions of interest. In this case, the a priori domains originated from the primary research questions as framed in the six round 1 items. Initial coding of the data consisted of reading the responses and identifying sections of the text that correspond to the a priori domains and developing new domains as needed. A primary analyst reviewed and cataloged each response. A secondary analyst reviewed the cataloged data and inserted commentary throughout, providing a second perspective and additional input. The qualitative team resolved discrepant coding through discussion. The qualitative team then distributed a summary of these results to the larger research team for further input. The broader research team agreed on the final set of domains after thorough review and debate. The team shared these domains with both the focus group participants and the Delphi panelists in the subsequent rounds. We describe the details of the focus group discussions elsewhere (forthcoming) including the use of digital recordings, transcription, coding, and memoing procedures.

Between rounds 1 through 3, the investigative team discussed the data and combined redundant topics, split multifactored topics, and eliminated topics. Typically, eliminated topics included those that the team agreed were correlates of engagement in care (eg, substance use, depression) or that the team had framed as outcomes of engagement in care (ie, retention in care, adherence to medications, viral suppression) rather than aspects, facets, or dimensions of engagement. Our a priori goal was to identify universal elements of engagement in care so that a set of items that are applicable to all patients would result. Therefore, we removed items that would not be applicable to all patients (eg, childcare needs) so that all patients would be able to complete the subsequent measure.

## Results

**Figure 2:**
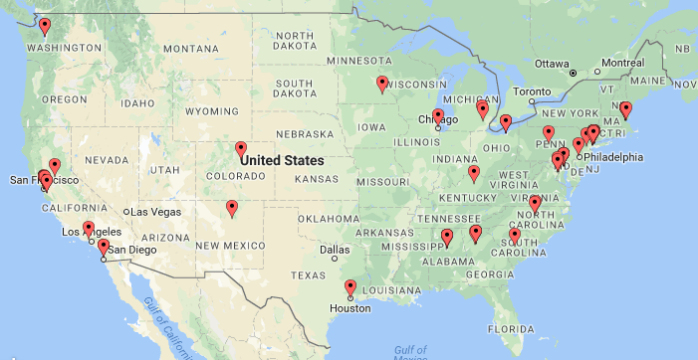
Geographic distribution of Delphi panelists.

A total of 108 experts were identified and invited to participate as Delphi panel members, which resulted in our exceeding the target of 20 for two of the three groups. Overall, 66 agreed to participate and enrolled (61.1% acceptance rate). The enrollment rate was higher for group 1 than the other groups, which is likely because those individuals had a documented interest in the topic, as evidenced by their publications and grant funding in the area. Of the 66 who began, 64 completed all four rounds (97%) with one person each in groups 1 and 2 failing to respond beyond the second Delphi round. See Table 1 for breakdown by Delphi group. Although we did not formally seek geographic variability in our recruitment of panelists, Figure 2 demonstrates that we had widespread geographic representation in the panelists. There was a mean of 7.75 (SD 1.54) patients in each of the 12 focus groups (N=93). Table 2 documents the characteristics of patients who participated in the focus groups as per the design in Figure 1. The time between Delphi rounds, focus groups, and cognitive interviews varied depending on the amount of analysis and logistical demands required for each task, with the overall data collection spanning 25 months concluding in June 2016.

As per our a priori design, the three groups reflected a range of research and clinical perspectives. See Table 3 for characteristics of the Delphi panelists overall and within each of the three groups.

As noted previously, we started with six broad questions in round 1. This led to 120 candidate topics, identified via Delphi panelists and patients in the focus groups, which formed the basis of round 2. We reduced these to 32 candidate items for round 3 and down to a final set of 13 items for round 4. The working definition of “engagement in care” that was developed prior to round 3 was as follows:

Engagement in care is the ongoing interaction of patients, their providers, and care settings that is characterized by a patient’s sense of connection to and active participation in care.

Panelists gave positive comments when asked about the process. Examples of comments included: “It has been a pleasure to be a part of such a meaningful project. Thank you for the opportunity!” and “This was an excellent process. As someone who works on the administering side of HIV research it was great to be a participant for a change. Each step was very clear and interesting,” and “Thanks for the great work! I’m excited to apply the findings from your study to my practice!”

**Table 1:** Response rates of participants.

Variable		Group 1: HIV engagement in care researchers	Group 2: HIV providers	Group 3: Non-HIV providers & researchers	Total
Invited, n		33	39	36	108
Enrolled, n (acceptance rate)		24 (73)	22 (56)	20 (56)	66 (61)
**Completed round, n (%)**					
	Round 1	24 (100)	22 (100)	20 (100)	66 (100)
	Round 2	24 (100)	22 (100)	20 (100)	66 (100)
	Round 3	23 (96)	21 (96)	20 (100)	64 (97)
	Round 4	23 (96)	21 (96)	20 (100)	64 (97)

**Table 2:** Focus group patient characteristics (N=93).

Variable		Participants
**Site, n (%)**		
	Birmingham	27 (29)
	San Francisco	30 (32)
	Seattle	36 (39)
Age in years (n=92), median (range)		49 (23-71)
Years since HIV diagnosis (n=92), median (range)		16 (1-38)
**Gender, n (%)**		
	Male	56 (60)
	Female	33 (35)
	Transgender	4 (5)
**Race, n (%)**		
	African American	41 (44)
	White	41 (44)
	Mixed race/other	11 (12)
**Ethnicity, n (%)**		
	Hispanic	10 (11)
	Non-Hispanic	83 (89)
**Injection drug use in past 12 months, n (%)**		
	No	76 (82)
	Yes	17 (18)
**Sexual orientation**^a^**, n (%)**		
	Bisexual	12 (14)
	Heterosexual	36 (41)
	Homosexual	40 (45)
**Currently on antiretroviral therapy**^b^**, n (%)**		
	No	11 (13)
	Yes	76 (87)
**Ever on antiretroviral therapy**^c^**, n (%)**		
	No	5 (5)
	Yes	87 (95)
**Detectable viral load**^d^**, n (%)**		
	No	55 (82)
	Yes	12 (18)

^a^Missing n=5 due to data collection oversight.

^b^Missing n=6 due to data collection oversight.

^c^Missing n=1 due to data collection oversight.

^d^Missing n=26 due to data collection oversight.

**Table 3:** Delphi panel characteristics.

Variable			Group 1: HIV engagement in care researchers (n=24)	Group 2: HIV providers (n=22)	Group 3: Non-HIV providers & researchers (n=20)	Total sample (N=66)
**Gender, n (%)**						
	Female		16 (67)	15 (68)	15 (75)	46 (70)
	Male		8 (33)	7 (32)	4 (20)	19 (30)
	Not disclosed		0	0	1 (5)	1 (2)
Age in years, mean (SD)			46.1 (7)	46.1 (9)	46.9 (10)	46.4 (8)
**Race and ethnicity, n (%)**						
	Hispanic/Latino(a)		1 (4)	0	2 (10)	3 (5)
	African American		2 (8)	3 (14)	2 (10)	7 (11)
	Asian		1 (4)	2 (9)	1 (5)	4 (6)
	White		20 (83)	15 (68)	13 (65)	50 (71)
	Native Hawaiian or other Pacific Islander		0	1 (5)	0	1 (2)
	Other or not specified		0	1 (5)	2 (10)	3 (5)
**Primary discipline, n (%)**						
	Medicine		10 (42)	12 (55)	12 (60)	34 (52)
	Social or behavioral science		8 (33)	0	6 (30)	16 (24)
	Public health		6 (25)	0	0	6 (9)
	Nursing		0	6 (27)	1 (5)	7 (11)
	Social work		0	4 (18)	1 (5)	5 (8)
	Other		0	0	0	5 (8)
**Academic rank, n (%)**						
	Instructor		1 (4)	1 (5)	1 (5)	3 (5)
	Assistant professor		3 (13)	3 (14)	9 (45)	15 (23)
	Associate professor		11 (46)	2 (9)	1 (5)	14 (22)
	Professor		4 (17)	3 (14)	7 (35)	14 (22)
	Other		2 (8)	6 (27)	1 (5)	9 (14)
	Not applicable (no academic appointment)		3 (13)	7 (32)	1 (5)	11 (17)
**Primary work setting, n (%)**						
	University		20 (83)	3 (14)	13 (65)	36 (54.6)
	Hospital/Clinic		0	19 (86)	4 (20)	23 (34.9)
	Government or public health dept.		3 (13)	0	0	3 (4.5)
	Other		1 (4)	0	3 (15)	4 (6.0)
**Direct patient care, n (%)**			9 (38)	22 (100)	15 (75)	46 (69)
	**Among those with patient care**				
		Outpatient only, n (%)	2 (22)	14 (64)	6 (40)	22 (48)
		Inpatient and outpatient, n (%)	7 (78)	8 (36)	9 (60)	24 (52)
		Patients with HIV seen per week, mean (SD)	10.6 (4.4)	52.4 (32.2)	1.0 (1.4)	27.4 (32.5)
		Patients without HIV seen per week, mean (SD)	1.3 (2.3)	4.0 (10.4)	15.7 (12.1)	7.3 (11.4)
		Total patient load (patients with HIV), mean (SD)	118.0 (96.9)	269.7 (126.9)	7.9 (12.5)	154.6 (150.5)
		Total patient load (patients without HIV), mean (SD)	11.9 (18.9)	41.0 (122.6)	198.2 (176.4)	85.6 (150.2)

## Discussion

The modified Delphi method of consensus used in this study emerged as an efficient method of gathering input across a group of experts separated by geography, discipline, expertise, and professional roles. This feature of the Delphi method allowed the solicitation of perspectives from busy professionals, whose schedules and competing demands would likely have limited their participation in more time-intensive research procedures. Sampling clinicians and researchers within and outside of HIV allowed a diverse set of complementary perspectives that enabled a comprehensive exploration of factors potentially relevant to engagement in HIV care. Researchers and clinicians are a rich source of information on topics such as engagement in care, but they may work in isolation or in closed networks; with the exception of presentations, case conferences, and specific publications often appearing in niche journals, the field may not systematically seek and integrate their perspectives. Bringing together experts through this Delphi process encouraged a wide range of complementary perspectives that served to illuminate many aspects of the construct in question that might otherwise have not been formally encouraged. For example, some participants prioritized clinical outcomes (eg, virologic suppression) as the only meaningful indicator of engagement in care, whereas others emphasized the importance of a strong patient-provider relationship as key to engagement in care. The process was relatively easy and input from panelists suggests that they found it to be engaging and interesting. Moreover, the online panel provided a way to obtain expert opinion that was less resource intensive than hosting an in-person meeting to convene geographically dispersed participants.

Our decision to include focus group discussions and cognitive interviews with patients reflects a departure from traditional use of the Delphi process, which typically seeks to restrict input to the members of the panel only. However, deliberately integrating patient perspectives into the process allowed for a more diverse set of perspectives than if patient data not been used. For example, it ensured that some topics that did not emerge from the experts were considered during analysis and transmitted to the Delphi panelists for their reactions. Indeed, we found patient perspectives critical to the success of our overarching objective to develop a patient-centered measure. Our design results in an innovative integration of Internet-based data collection procedures with in-person data collection that provides a convergence of perspectives that otherwise would be difficult to achieve.

The process allowed for a rich broadening and expanding of content in response to open-ended questions in round 1 and then a subsequent reduction and refining of content in later rounds. The final set of 13 items reflects the input of all 66 Delphi experts from around the United States and patients in three cities. Illustrative examples of the final items include the following two items: “How much of a role do you have in making decisions about your HIV care?” and “How comfortable do you feel asking questions during your HIV care appointments?” We intend to make the full-item list available once we have completed the initial validation of the index.

Although we did careful searching to identify members of the panel for the Delphi process, it is possible that some expertise areas, geographic regions, and disciplines were not as well represented as others. Although there are no standard metrics for enrollment in Delphi studies, the response rates to our invitations were varied across the three groups of experts. Similarly, we only conducted patient focus groups in three cities, which may limit the breadth of content than had we conducted focus groups in a larger number of geographic areas. Because we only included Delphi panelists and patients in the United States, findings may have limited generalizability to other countries and regions, where the array of factors associated with engagement in HIV care are likely different. Finally, because we chose to focus on universal aspects of engagement in care that would be relevant to all patients, we elected to exclude potential items relevant only for some subgroups, such as childcare and substance use treatment. Although this may improve the applicability across all patients, it may sacrifice some precision for specific groups for whom these targeted topics may be salient.

We are currently administering the final Index of Engagement to patients in care at seven HIV clinics across the United States. We anticipate collecting survey data from approximately 3000 patients, for whom we will be able to validate the index. This validation will include how well scores relate to factors we hypothesize would be correlated with engagement (eg, depression, HIV stigma, substance use) and outcomes/consequences of engagement (eg, self-reported medication adherence, retention in care, virologic suppression). As part of the process, we will distribute findings directly to the Delphi panelists; these will be in the form of reports, published papers, and slides from presentations. Depending on the outcomes of those validation analyses, we will explore developing interventions that target the predictive factors from the Index of Engagement to improve outcomes for people living with HIV.

## Abbreviations

HIV: human immunodeficiency virus
